# Supplementary material for: Ventilation-Associated Particulate Matter Is a Potential Reservoir of Multidrug-Resistant Organisms in Health Facilities
Source: Life (Basel). 2021 Jun 30;11(7):639. doi: 10.3390/life11070639 (PMC8307074; doi:10.3390/life11070639)
Supplement: Supplementary file 1 [file life-11-00639-s001.zip › life-1257405-supplementary.pdf]

life

MDPI

Article

Ventilation-Associated Particulate Matter is a Potential Reservoir of Multidrug-Resistant Organisms in Health Facilities

Evgenia Chezganova [1], Olga Efimova [2], Vera Sakharova [3], Anna Efimova [4], Sergey Sozinov [2], Anton Kutikhin [3], Zinfer Ismagilov [2] and Elena Brusina [1,*]

[1] Department of Epidemiology, Kemerovo State Medical University, Kemerovo 650056, Russia; chezganova@kemsma.ru
[2] Institute of Coal Chemistry and Material Science, Siberian Branch of the Russian Academy of Sciences, Kemerovo 650099, Russia; efimova@iccms.sbras.ru (O.E.); sozinov@iccms.sbras.ru (S.S.); ismagilov@iccms.sbras.ru (Z.I.)
[3] Department of Experimental Medicine, Research Institute for Complex Issues of Cardiovascular Diseases, Kemerovo 650002, Russia; sahavm@kemcardio.ru (V.S.); kytiag@kemcardio.ru (A.K.)
[4] Kemerovo Regional Center for Hygiene and Epidemiology, Kemerovo 650002, Russia; efimova@fguzko.ru
* Correspondence: brusina@kemsma.ru; Tel.: +7-905-962-18-15

Abstract: Most healthcare-associated infections (HCAIs) develop due to the colonisation of patients and healthcare workers by multidrug-resistant organisms (MDRO). Here, we investigated whether the particulate matter from the ventilation systems (Vent-PM) of health facilities can harbour MDRO and other microbes, thereby acting as a potential reservoir of HCAIs. Dust samples collected in the ventilation grilles and adjacent air ducts underwent a detailed analysis of physico-chemical properties and biodiversity. All Vent-PM samples included ultrafine PM capable of reaching the alveoli. Strikingly, > 70% of Vent-PM samples were contaminated, mostly by viruses (> 15%) or multidrug-resistant and biofilm-producing bacterial strains (60% and 48% of all bacteria-contaminated specimens, respectively). Total viable count at 1 m from the ventilation grilles was significantly increased after opening doors and windows, indicating an association between air flow and bacterial contamination. Both chemical and microbial compositions of Vent-PM considerably differed across surgical vs. non-surgical and intensive vs. elective care units and between health facilities located in coal and chemical districts. Reduced diversity among MDRO and increased prevalence ratio in multidrug-resistant to the total *Enterococcus spp.* in Vent-PM testified to the evolving antibiotic resistance. In conclusion, we suggest Vent-PM as a previously underestimated reservoir of HCAI-causing pathogens in the hospital environment.

Keywords: particulate matter; air pollution; hospital dust; ventilation grilles; multidrug-resistant organisms; healthcare-associated infections; HCAI-causing pathogens; reservoir of infection; microbial diversity; chemical composition

Citation: Chezganova, E.; Efimova, O.; Sakharova, V.; Efimova, A.; Sozinov, S.; Kutikhin, A.; Ismagilov, Z.; Brusina, E. Ventilation-Associated Particulate Matter is a Potential Reservoir of Multidrug-Resistant Organisms in Health Facilities. *Life* **2021**, *11*, 639. https://doi.org/10.3390/life11070639

Academic Editor: Milan Kolář

Received: 27 May 2021
Accepted: 29 June 2021
Published: 30 June 2021

Publisher's Note: MDPI stays neutral with regard to jurisdictional claims in published maps and institutional affiliations.

Copyright: © 2021 by the authors. Licensee MDPI, Basel, Switzerland. This article is an open access article distributed under the terms and conditions of the Creative Commons Attribution (CC BY) license (http://creativecommons.org/licenses/by/4.0/).

Supplementary Materials:

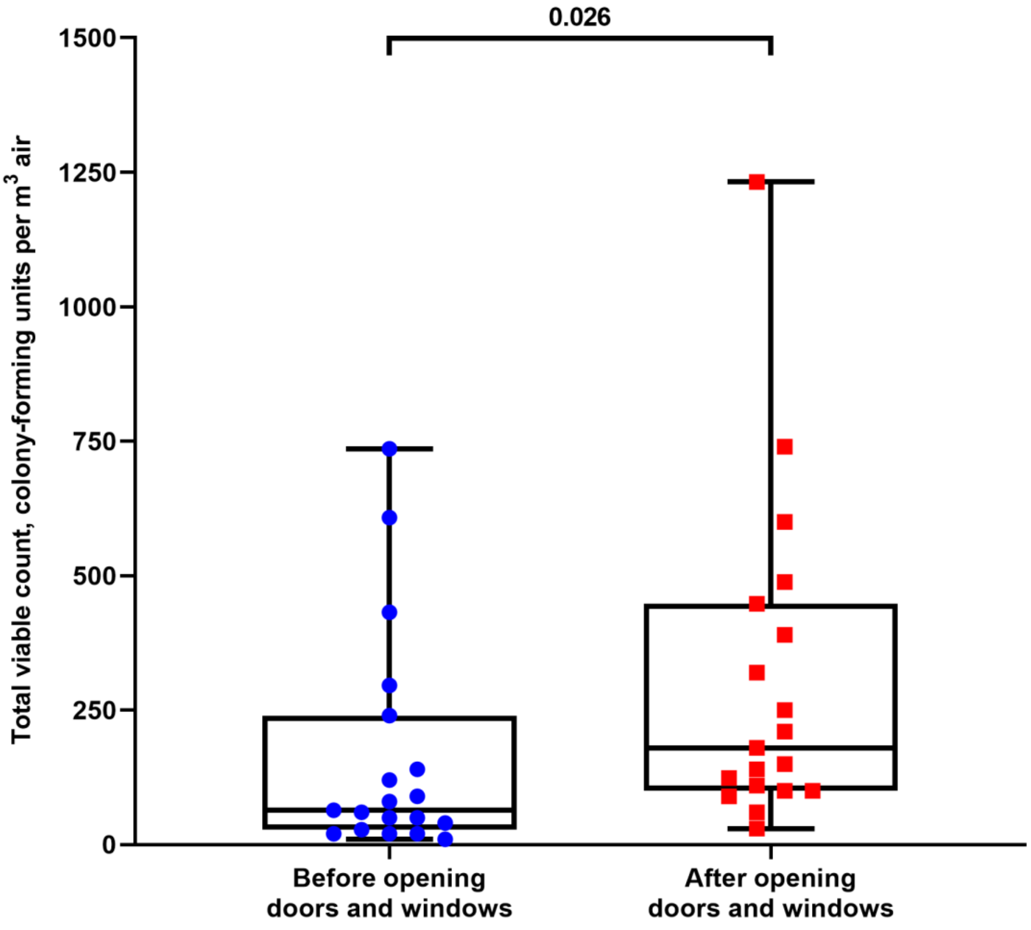

Figure S1. Total viable count in the hospital air before (blue circle dots) and after (red square dots) opening doors and windows. Each dot represents one air sample. *P* values provided above the boxes, Wilcoxon matched-pairs signed rank test.
